# Adherence to Tuberculosis Treatment, Sputum Smear Conversion and Mortality: A Retrospective Cohort Study in 48 Rwandan Clinics

**DOI:** 10.1371/journal.pone.0073501

**Published:** 2013-09-16

**Authors:** Felix R. Kayigamba, Mirjam I. Bakker, Veronicah Mugisha, Ludwig De Naeyer, Michel Gasana, Frank Cobelens, Maarten Schim van der Loeff

**Affiliations:** 1 INTERACT, Kigali, Rwanda; 2 Royal Tropical Institute, KIT Biomedical Research, Amsterdam, The Netherlands; 3 ICAP, Mailman School of Public Health, Columbia University, Kigali, Rwanda; 4 National Tuberculosis Control Program in Rwanda (PNILT), Kigali, Rwanda; 5 Amsterdam Institute for Global Health and Development (AIGHD), Amsterdam, The Netherlands; 6 Department of Global Health, Academic Medical Center (AMC), Amsterdam, The Netherlands; 7 Center for Infection and Immunity Amsterdam (CINIMA), Academic Medical Center (AMC), Amsterdam, The Netherlands; 8 Public Health Service of Amsterdam (GGD), Department of Infectious Diseases, Amsterdam, The Netherlands; Institute of Infectious Diseases and Molecular Medicine, South Africa

## Abstract

**Background:**

Adherence to treatment and sputum smear conversion after 2 months of treatment are thought to be important for successful outcome of tuberculosis (TB) treatment.

**Methods:**

Retrospective cohort study of new adult TB patients diagnosed in the first quarter of 2007 at 48 clinics in Rwanda. Data were abstracted from TB registers and individual treatment charts. Logistic regression analysis was done to examine associations between baseline demographic and clinical factors and three outcomes adherence, sputum smear conversion at two months, and death.

**Results:**

Out of 725 eligible patients the treatment chart was retrieved for 581 (80%). Fifty-six (10%) of these patients took <90% of doses (defined as poor adherence). Baseline demographic characteristics were not associated with adherence to TB treatment, but adherence was lower among HIV patients not taking antiretroviral therapy (ART); p = 0.03). Sputum smear results around 2 months after start of treatment were available for 220 of 311 initially sputum-smear-positive pulmonary TB (PTB+) patients (71%); 175 (80%) had achieved sputum smear conversion. In multivariable analysis, baseline sputum smear grade (odds ratio [OR] = 2.7, 95% Confidence interval [CI] 1.1–6.6 comparing smear 3+ against 1+) and HIV infection (OR 3.0, 95%CI 1.3–6.7) were independent predictors for non-conversion at 2 months. Sixty-nine of 574 patients (12%) with known TB treatment outcomes had died. Besides other known determinants, poor adherence had an independent, strong effect on mortality (OR 3.4, 95%CI 1.4–7.8).

**Conclusion:**

HIV infection is an important independent predictor of failure of sputum smear conversion at 2 months among PTB+ patients. Poor adherence to TB treatment is an important independent determinant of mortality.

## Introduction

Tuberculosis (TB) is a main cause of morbidity and mortality, especially in resource-poor countries. According to the 2011 World Health Organization (WHO) Global TB Control report, 8.8 million new cases and 1.5 million deaths from TB occurred in 2010 [1]. Success of TB control rests on detection of new cases of TB (for which the WHO has set a target of 70%) and on successful treatment of those diagnosed (WHO target: 85%) [2]. Crucial to achieving a successful treatment outcome is adherence to treatment [3]. TB treatment usually lasts 6 or 8 months and low adherence to treatment, including defaulting from treatment, is one of the major challenges for control programs [4]–[6]. Among sputum smear-positive pulmonary TB (PTB+) patients, smear conversion after two months of intensive phase treatment is an important predictor for bacteriological cure [7], [8]. Poor adherence to treatment may prolong infectiousness and increases the risk of drug resistance, relapse, and death, as well as onward transmission [3], [9].

Rwanda is a densely populated country with an estimated annual TB incidence rate of 376 per 100,000 population [10]. The case detection rate is estimated to be only 19% [10]. In 2010 the number of newly notified TB patients was 6,434 [1]. Out of the 4165 new PTB+ patients 85% had a favorable outcome [1]. The HIV prevalence in the general population aged 15–49 years in Rwanda is 3.0% [11], and it is estimated that one third of TB cases are HIV infected [12]. Little is known about mortality of patients on TB treatment in Rwanda, and to what extent this is affected by TB treatment adherence.

In this retrospective cohort study, we examined the determinants of the following three outcomes: adherence to TB treatment, sputum smear conversion at two months, and TB mortality. We also examined the independent effect of adherence on mortality.

## Methods

### Diagnosis of TB

The diagnosis of pulmonary TB (PTB) in Rwanda is based on sputum smear microscopy. Any person who is coughing for more than 2 weeks should be considered a TB suspect and be invited for sputum smear examinations. During the time of this study, the Kinyoun staining technique was used [13]; in 2009, Ziehl-Neelsen (ZN) staining was adopted [14]. At the time of study, a diagnosis of PTB+ was made when at least 1 acid-fast bacillus (AFB) was observed in 100 fields, in at least 2 out of 3 smears, according to WHO guidelines (in Rwanda this criterion was later changed to a single positive smear qualifying as PTB+ in accordance with revised WHO guidelines [14]. The smear is graded according to the number of AFBs found: scanty, 1+, 2+, or 3+ [13]. If all three sputum smears are negative, but there is strong supportive evidence for TB, a diagnosis of smear-negative PTB can be made. In Rwanda it is a policy to test TB patients for HIV. Those found to be HIV infected are referred for CD4 count testing.

### Treatment of TB

The treatment regimen for new cases of TB in Rwanda consists of a two month intensive phase with a daily dose of fixed-dose combination tablets containing rifampicin (R), isoniazid (H), pyrazinamide (Z) and ethambutol (E), followed by a four months continuation phase of daily RH. To monitor treatment response of smear-positive cases, sputum smear microscopy is done after the second (C2), fourth and sixth month of treatment. PTB+ cases are expected to convert to negative sputum smear status after the two months of intensive treatment. Patients whose sputum smear has not converted after two months of treatment are prescribed an additional month of intensive phase treatment. The National Tuberculosis Control Program in Rwanda (PNILT) follows the WHO standard definitions of the six treatment outcomes (cure, treatment completion, death, treatment failure, default, transferred-out) [8].

### Study Design

We conducted a retrospective cohort study of TB patients in 3 of the 5 provinces of Rwanda. Kigali Province, which is predominantly urban, and the Western and Northern Provinces, which are mostly rural. From Kigali and the Northern provinces all districts were included while one district (Ngororero) from the Western province was included. Provinces and districts were purposefully chosen to include both rural and urban areas, and both central and peripheral areas. Patients with all forms of new TB (PTB+, smear-negative PTB, PTB of unknown smear status, and extrapulmonary TB [EPTB]), registered in all TB diagnostic facilities (n = 48) from the selected districts in the first quarter of 2007 were included in the study. Patients less than 15 years of age and those classified as previously treated cases were excluded. Data of all eligible patients were abstracted in November and December 2007 from the TB registers at the health facilities by trained data collectors, using a standardised form. These data were used to retrieve TB treatment charts for individual patients in the period February to August 2008. Treatment charts are used by treating physicians, nurses or other medical personnel to indicate details of the individual patient’s condition, treatment prescribed and treatment taken, and treatment outcomes. The patient treatment charts were abstracted to obtain demographic information, details of TB diagnosis (including sputum smear results), HIV test results and treatment, information on number of daily doses taken, and treatment outcome. CD4 count data were very incomplete and not done at standardised moments, and were not included in the analyses.

### Study Outcomes

The outcomes of interest in this study included: (1) adherence to TB treatment; (2) sputum smear conversion after two months of TB treatment (among PTB+ patients only); (3) mortality during TB treatment.

Adherence to TB treatment was defined as good if 90% or more of expected TB medication doses were taken, and defined as poor if less than 90% of the doses were taken [15]. The expected number of doses was calculated as the number of days between the start of treatment and the date of treatment completion, or date of death, default, or treatment failure, as appropriate. Thus, even patients who died within six months of start of TB treatment could achieve 100% adherence as only the period that they were alive was included in the denominator. Also, patients who took 168 doses (i.e. the number of doses to complete treatment: 6 months times 28 days) of treatment could be classified as non-adherent, if they needed more than 187 days (168/187 = 90%) to complete their treatment (due to interruptions). Patients who were transferred out were excluded from this analysis, as data on doses taken were very incomplete in this group.

Sputum smear conversion after two months was defined as a negative smear result from a sputum sample that was obtained at least 7 weeks and not more than 9 weeks after the start of the TB treatment in patients diagnosed with PTB+.

### Ethics

This was a retrospective study making use of data already collected; all data used in this report were routine clinical data collected in the process of diagnosis and treatment. The processing and analysis of data was done after anonymization. Therefore no informed consent was requested from patients. This approach was reviewed and approved by the Rwanda National Ethics Committee and the Research Ethics Committee of the Academic Medical Center (AMC), Amsterdam, the Netherlands. The funders of the study had no role in the analysis or the decision to publish the data.

### Statistical Analysis

Data were double entered into a SQL server platform database (Microsoft, USA) and analysed using Stata version 11 (Stata Corporation, College Station, TX, USA). We examined the association of various baseline characteristics on the three study outcomes (i.e. adherence; sputum smear conversion at C2; death). Differences between categories were analysed using the chi-squared test or Fisher’s exact test, as appropriate. The distributions of continuous variables (these were not normally distributed) were compared between groups using the rank sum test.

Logistic regression was used to identify factors that were independently associated with the study outcome measures. As we examined relatively few baseline characteristics (sex, age group, occupation, type of TB, HIV status, use of antiretroviral treatment (ART), use of co-trimoxazole prophylaxis, weight, and location of facility) these were all included in a starting model. In the analyses use of ART or co-trimoxazole prophylaxis was defined as being on ART or co-trimoxazole prophylaxis before starting TB treatment. Using the likelihood ratio test, we eliminated variables one by one from the model (if p>0.05) until a parsimonious model was obtained. Sex and age group were forced into the models, because they were a priori thought to have a major impact on outcomes. P values below 0.05 were considered significant.

## Results

In the first quarter of 2007 a total of 725 new TB patients fulfilling the eligibility criteria were identified from the TB registers in the 48 facilities. From 581 of these 725 the treatment charts were retrieved (80%). The 144 patients of whom the treatment card could not be retrieved were not different in terms of age and failure to convert at C2, but tended to be more often female (47% vs. 38%; p = 0.052); they more often had EPTB (40% vs. 28%; p = 0.027), and their treatment outcome was significantly more often death (21% vs. 12%; p = 0.003) or transfer out (51% vs. 3%; p<0.001). Our subsequent analyses are based on the 581 patients of whom the treatment card could be retrieved and are thus based on a population with, on average, better TB treatment outcomes.

The number of new TB patients per health facility varied between 0 and 55 (median 4.5); from seven clinics no patients were included in the study. In Table 1 the baseline characteristics of the 581 patients are summarised. Sixty-two percent were male, and the median age was 31 years (range 15–86 years). Forty-two percent of patients were HIV infected at the time of TB diagnosis. Among the PTB+ patients 29% (90/311) were HIV infected, and among all other TB patients 55% (145/262; p<0.001). Thirty percent (73/242) of HIV infected patients had started ART by the time of the start of TB treatment; an additional 37 (15%) started ART within the first three months of TB treatment.

**Table 1:** Baseline characteristics and follow-up data of 581 patients with tuberculosis, Rwanda, 2007.

	N (%) or median (IQR)
***A - Baseline characteristics***	
**1. Demographic factors**	
**Sex**	
Male	359 (61.8)
Female	222 (38.2)
**Age (years)**	
Median age (IQR)	31 (25–41)
15–24	145 (25.0)
25–34	199 (34.3)
35–44	126 (21.7)
≥45	111 (19.1)
**Occupation**	
Low-income group*	193 (33.2)
Students	44 (7.6)
Prisoners	23 (4.0)
Employed	105 (18.1)
Missing	216 (37.2)
**2. Clinical factors**	
**Type of TB**	
PTB scanty or 1+	109 (18.8)
PTB 2+	109 (18.8)
PTB 3+	93 (16.0)
PTB smear neg	64 (11.0)
PTB “microscopy not-done”	34 (5.9)
Extra-pulmonary TB	164 (28.2)
Missing	8 (1.4)
**HIV**	
Negative	327 (56.3)
Positive	242 (41.7)
Missing	12 (2.1)
**Weight (kgs)**	
Median weight (IQR)	51 (45–58)
≤48	208 (35.8)
49–55	177 (30.5)
≥56	175 (30.1)
Missing	21 (3.6)
**3. Treatment centre factors**	
**Location of health facility**	
Rural	68 (11.7)
Urban	513 (88.3)
**District**	
Burera	13 (2.2)
Gakenke	24 (4.1)
Gasabo	143 (24.6)
Gicumbi	26 (4.5)
Kicukiro	104 (17.9)
Musanze	58 (10.0)
Ngororero	17 (2.9)
Nyarugenge	196 (33.7)
***B. Follow-up data***	
**Adherence**	
Took ≥90% of doses	480 (82.6%)
Took <90% of doses	56 (9.6%)
No data	45 (7.8%)
**Repeat C2 sputum smear status** **	
Negative sputum smear	226 (72.7%)
Positive sputum smear	52 (16.7%)
No sputum smear result	33 (10.6%)
**Antiretroviral treatment**	
Not HIV infected	327 (56.3%)
HIV infected, on ART at time of start TB R/	73 (12.6%)
HIV infected, started ART within 3 mo of start TB R/	37 (6.4%)
HIV infected, not on ART or started >3 mo after start of TB R/	132 (22.7%)
HIV status unknown, no ART	12 (2.1%)
**Use of co-trimoxazole prophylaxis**	
Not HIV infected	327 (56.3%)
HIV infected, on co-trimoxazole at time of start TB R/	107 (18.4%)
HIV infected, started co-trimoxazole within 3 mo of start TB R/	48 (8.3%)
HIV infected, not on co-trimoxazole or started >3 mo after start of TB R/	87 (15.0%)
HIV status unknown, no co-trimoxazole	12 (2.1%)
**Outcome**	
Cured	236 (40.6%)
Treatment completed	221 (38.0%)
Treatment failure	15 (2.6%)
Died	69 (11.9%)
Defaulted	13 (2.2%)
Transfer out	20 (3.4%)
Outcome not recorded	7 (1.2%)

Abbreviations: ART antiretroviral treatment; TB Tuberculosis; PTB pulmonary TB; IQR inter-quartile range; HIV Human Immunodeficiency Virus; kg kilogram.

* Farming, housewives, unemployed, laborer, vendor;

** smear status 2 months after start of TB treatment (for details see text); percentages based on those with smear positive PTB at baseline.

### Associations between Baseline Characteristics and ART, and TB Treatment Adherence

Information on adherence to TB treatment was available for 536 patients. In total, 480 (90%) patients took 90% or more of expected medication doses of TB treatment; 56 (10%) took <90% of expected doses. In univariate analysis age nor sex were associated with poor adherence (Table 2). There was some evidence for association of HIV status with poor adherence (OR 1.7; 95%CI 0.97–3.0). A combined variable of HIV status and antiretroviral treatment was significantly associated with adherence: those who were HIV infected but not on ART were significantly more often poorly adherent (OR 2.4; 95% CI 1.1–3.7). In multivariable analysis using logistic regression, adjusting for age and sex, only untreated HIV was significantly associated with poor adherence (OR 2.4, 95%CI 1.2–4.6). Addition of any of the other variables did not change the independent effect of untreated HIV (data not shown). As this association might have been artificially created by those who died during the TB treatment having been more often recorded as non-adherent, we repeated the analysis restricted to patients who did not die. In that analysis, based on 463 patients, the independent effect of untreated HIV status on adherence was even stronger (OR 2.8, 95%CI 1.3–5.9).

**Table 2:** Association of demographic and clinical characteristics with poor adherence in 536 newly identified TB patients, Rwanda, 2007.

			Univariate analysis			Multivariable analysis		
Baseline characteristics	N	n (%) withpoor adherence	OR	95% CI	P	OR	95% CI	P
**Sex**								
Male	337	39 (11.6)	1		0.26	1		0.24
Female	199	17 (8.5)	0.71	0.39–1.30		0.69	0.37–1.29	
**Age (years)**								
15–24	129	14 (10.9)	1		0.99	1		0.74
25–34	187	19 (10.2)	0.93	0.45–1.93		0.74	0.34–1.59	
35–44	119	12 (10.1)	0.92	0.41–2.08		0.60	0.24–1.49	
≥45	101	11 (10.9)	1.00	0.43–2.32		0.75	0.31–1.85	
**Occupation**								
Low-income group*	176	21 (11.9)	1		0.56			
Students	41	2 (4.9)	0.38	0.09–1.68				
Prisoners	21	0 (0.0)	–					
Employed	99	11 (11.1)	0.92	0.43–2.00				
Not recorded	199	22 (11.1)	0.92	0.49–1.73				
**Type of TB**								
PTB scanty or 1+	104	5 (4.8)	1		0.16			
PTB 2+	102	15 (14.7)	3.41	1.19–9.78				
PTB 3+	80	8 (10.0)	2.20	0.69–7.00				
PTB smear neg	60	8 (13.3)	3.05	0.95–9.78				
PTB “microscopy not-done”	30	5 (16.7)	3.96	1.06–14.75				
Extra-pulmonary TB	152	14 (9.2)	2.01	0.70–5.76				
Not recorded	8	1 (12.5)						
**HIV status**								
Negative	301	25 (8.3)	1		0.06			
Positive	225	30 (13.3)	1.70	0.97–2.98				
Not tested	10	1 (10.0)						
**Weight (kgs)**								
≤48	188	20 (10.6)	1		0.77			
49–55	170	18 (10.6)	0.99	0.51–1.95				
≥56	163	14 (8.6)	0.79	0.39–1.62				
Not recorded	15	4 (26.7)						
**Location of health facility**								
Rural	63	6 (9.5)	1		0.80			
Urban	473	50 (10.6)	1.12	0.46–2.74				
**District**								
Burera	13	1 (7.7)	1		0.75			
Gakenke	21	1 (4.8)	0.60	0.03–10.51				
Gasabo	119	14 (11.8)	1.60	0.19–13.26				
Gicumbi	26	5 (19.2)	2.86	0.30–27.41				
Kicukiro	999	12 (12.1)	1.66	0.20–13.89				
Musanze	54	4 (7.4)	0.96	0.10–9.39				
Ngororero	17	2 (11.8)	1.60	0.13–19.84				
Nyarugenge	187	17 (9.1)	1.20	0.15–9.80				
**Antiretroviral therapy** 1								
Not HIV infected	301	25 (8.3)	1		0.06	1		0.035
HIV infected, on ART	70	6 (8.6)	1.04	0.41–2.63		1.25	0.47–3.21	
HIV infected, not on ART	155	24 (15.5)	2.02	1.11–3.68		2.36	1.22–4.55	
Unknown HIV status	10	1 (10.0)						
**Co–trimoxazole prophylaxis** 1								
Not HIV infected	301	25 (8.3)	1		0.17			
HIV+, on co-trimoxazole	103	13 (12.6)	1.59	0.78–3.25				
HIV+, not on co-trimoxazole	122	17 (13.9)	1.79	0.93–3.44				
Unknown HIV status	10	1 (10.0)						

* Farming, housewives, unemployed, laborer, vendor.

ART antiretroviral therapy, OR Odds Ratio, aOR ajusted Odds Ratio, CI Confidence Interval, PTB Pulmonary TB.

1 On ART/co-trimoxazole: on this treatment before or at the time of start of TB treatment; not on ART/co-trimoxazole: started with this treatment after starting TB treatment or not at all.

### Associations between Baseline Characteristics and Sputum Smear Conversion among PTB+ Patients

Out of 311 initially PTB+ patients, 292 were still on treatment two months after start of treatment. A control smear result (irrespective of timing) was available for 278 (95.2%) of these patients, at a median (IQR) of 57 days (54–61) after start of treatment. Time since start of TB treatment was a significant predictor of smear conversion (OR for non-conversion for each additional day since start of TB treatment: 0.94 (95%CI 0.89–0.99). For 220 (79%) a C2 smear was done between 7 and 9 weeks after start of TB treatment, and 175 (80%) of these had achieved sputum smear conversion. In univariate analysis, those with a smear grade of 3+ at baseline (OR 2.4, 95%CI 1.02–5.7) and those with HIV infection (OR 2.5, 95%CI 1.3–5.1) were more likely to remain sputum smear-positive than patients with smear grades 1+ or 2+ at baseline and those not HIV infected, respectively (Table 3). In a multivariable logistic regression analysis adjusting for age and sex, independent predictors for non-conversion at 2 months were baseline sputum smear grade (OR = 2.7, 95% CI 1.1–6.6 for comparing those with smear 3+ against those with smear 1+), and HIV infection (OR = 3.0, 95%CI 1.3–6.7). A logistic model that included ART/HIV status combined rather than HIV status was not significantly better (likelihood ratio test, p = 0.22). Addition of any of the other variables did not change the independent effect of HIV (data not shown).

**Table 3:** Association of sputum smear conversion after 2 months of intensive treatment phase with baseline characteristics in 220 smear-positive PTB patients, who had a sputum sample taken between 7 and 9 weeks after start of treatment, Rwanda 2007.

Baseline characteristics	N	n (%) withoutsputum conversionafter 2 months	Univariate analysis			Multivariable analysis†		
			OR	95% CI	P	OR	95% CI	P
**Sex**								
Male	156	35 (22.4%)	1		0.25	1		0.24
Female	64	10 (15.6%)	0.64	0.30–1.39		0.61	0.27–1.39	
**Age (years)**								
15–24	66	12 (18.2%)	1		0.45	1		0.19
25–34	81	14 (17.3%)	0.94	0.40–2.20		0.53	0.21–1.36	
35–44	40	9 (22.5%)	1.31	0.49–3.45		0.68	0.22–2.10	
≥45	33	10 (30.3%)	1.96	0.74–5.17		1.39	0.48–4.01	
**Occupation**								
Low-income group*	88	26 (29.6%)	1		0.12			
Students	21	3 (14.3%)	0.40	0.11–1.47				
Prisoner	10	0 (0%)	–					
Employed	43	7 (16.3%)	0.46	0.18–1.18				
Not recorded	58	9 (15.5%)	0.44	0.19–1.02				
**Smear grade category**								
PTB scanty or 1+	73	10 (13.7%)	1		0.12	1		0.087
PTB 2+	82	17 (20.7%)	1.65	0.70–3.87		1.54	0.63–3.77	
PTB 3+	65	18 (27.7%)	2.41	1.02–5.70		2.69	1.09–6.63	
**HIV status**								
Negative	159	25 (15.7%)	1		0.009	1		0.008
Positive	59	19 (32.2%)	2.55	1.27–5.09		2.99	1.33–6.71	
Not tested	2	1 (50.0%)						
**Weight (kgs)**								
≤48	79	19 (24.1%)	1		0.63			
49–55	73	13 (17.8%)	0.68	0.31–1.51				
≥56	64	13 (20.3%)	0.80	0.36–1.79				
Not recorded	4	0 (0%)						
**Location of health facility**								
Rural	45	11 (24.4%)	1		0.46			
Urban	175	34 (19.4%)	0.75	0.34–1.62				
**District**								
Burera	7	2 (28.6%)	1		0.059			
Gakenke	20	8 (40.0%)	1.67	0.26–10.8				
Gasabo	40	8 (20.0%)	0.63	0.10–3.83				
Gicumbi	16	1 (6.3%)	0.17	0.01–2.26				
Kicukiro	42	5 (11.9%)	0.34	0.05–2.22				
Musanze	14	2 (14.3%)	0.42	0.05–3.83				
Ngororero	10	5 (50.0%)	2.50	0.32–19.5				
Nyarugenge	71	14 (19.7%)	0.61	0.11–3.50				
**Antiretroviral treatment** #								
Not HIV infected	159	25 (15.7)	1		0.017			
HIV+, not on ART	46	13 (28.3)	2.11	0.98–4.56				
HIV+, on ART	13	6 (46.2)	4.59	1.42–14.8				
Unknown HIV status	2							
**Co-trimoxazole prophylaxis** #								
Not HIV infected	159	25 (15.7)	1		0.026			
HIV+, not on co-trimoxazole	38	11 (29.0)	2.18	0.96–4.96				
HIV+, on co-trimoxazole	21	8 (38.1)	3.30	1.24–8.78				
Unknown HIV status	2							

† Multivariate analysis was based on n = 218.

* Farming, housewives, unemployed, labourer, vendor.

# On ART/Co-trimoxazole: already on these treatments at the time of start of TB treatment. Not ART/Co-trimoxazole: started with these treatments after start of TB treatment or did not start at all before end of treatment.

OR - Odds Ratios; CI - Confidence Interval, PTB Pulmonary TB, ART aniretroviral treatment.

We re-examined this association in a model in which we included patients with a C2 done before 7 weeks after start of TB treatment whose C2 was already negative, and also patients with a C2 done more than 9 weeks after start of TB treatment whose C2 was still positive. In this analysis, based on 228 patients, HIV was still an independent predictor of smear conversion (OR 2.8, 95%CI 1.3–6.2).

### Associations between Baseline Characteristics and Mortality

TB treatment outcome was registered for 574 patients: 236 (41%) were cured, 221 (39%) had completed treatment, 15 (3%) had treatment failure, 69 (12%) had died, 13 (2%) had defaulted and 20 (3%) had been transferred out. Of the 69 deaths, 41 occurred among HIV-infected patients. The following factors were associated with mortality in univariate analysis: age, occupation, type of TB, HIV status, district, antiretroviral treatment, and co-trimoxazole prophylaxis (Table 4). In a multivariable analysis only age (OR = 4.7, 95% CI 1.9–11.9, for age≥45 years compared to age 15–24), HIV infection (OR = 1.9, 95% CI 1.01–3.5, compared to HIV-negative patients), and type of TB were significantly associated with death (Table 4). Patients with smear-negative PTB (OR 7.5, 95%CI 1.5–36.7) and those with EPTB (OR 11.5, 95%CI 2.6–50.3) were significantly more likely to have died, compared to the patients with scanty or 1+ positive PTB.

**Table 4:** Associations of mortality during TB treatment with demographic and clinical characteristics in 574 TB patients, Rwanda, 2007.

			Univariate analysis			Multivariable analysis		
Baseline characteristics	N	n (%) who died	OR	95% CI	P	aOR	95% CI	P
**Sex**								
Male	353	38 (10.8%)	1		0.25	1		0.82
Female	221	31 (14.0%)	1.35	0.81–2.25		1.07	0.59–1.96	
**Age (years)**								
15–24	144	7 (4.9%)	1		<0.0001	1		0.0002
25–34	196	16 (8.2%)	1.74	0.70–4.35		1.06	0.40–2.84	
35–44	125	17 (13.6%)	3.08	1.23–7.70		1.60	0.58–4.44	
≥45	109	29 (36.6%)	7.09	2.97–16.94		4.72	1.88–11.87	
**Occupation**								
Low-income group*	191	19 (10.0%)	1		0.024			
Students	43	0 (0.0%)	–	–				
Prisoners	23	3 (13.0%)	1.36	0.37–5.00				
Employed	104	8 (7.7%)	0.75	0.32–1.79				
Not recorded	213	39 (18.3%)	2.03	1.13–3.65				
**Type of TB**								
PTB smear scanty or 1+	108	2 (1.9%)	1		<0.0001	1		<0.0001
PTB smear 2+	108	4 (3.7%)	2.04	0.37–11.37		1.54	0.25–9.53	
PTB smear 3+	91	7 (7.7%)	4.42	0.89–21.82		3.95	0.78–19.97	
PTB smear neg.	63	11 (17.5%)	11.21	2.40–52.44		7.50	1.53–36.70	
PTB “microscopy not-done”	33	2 (6.1%)	3.42	0.46–25.28		1.26	0.11–14.87	
Extra-pulmonary TB	163	39 (23.9%)	16.67	3.93–70.67		11.49	2.63–50.25	
Not recorded	8	4 (50.0%)						
**HIV status**								
Negative	323	23 (7.1%)	1		0.0002	1		0.043
Positive	240	41 (17.1%)	2.69	1.56–4.62		1.88	1.01–3.51	
Not tested	11	5 (45.5%)						
**Weight (kg)**								
≤48	204	28 (13.7%)	1		0.45			
49–55	176	19 (10.8%)	0.76	0.41–1.42				
≥56	175	17 (9.7%)	0.68	0.36–1.28				
Not recorded	19	5 (26.3%)						
**Location of health facility**								
Rural	68	5 (7.4%)	1		0.18			
Urban	506	64 (12.7%)	1.82	0.71–4.71				
**District**								
Burera	13	3 (23.8%)	1		0.022			
Gakenke	24	2 (8.3%)	0.30	0.04–2.11				
Gasabo	141	11 (7.8%)	0.28	0.07–1.18				
Gicumbi	26	1 (3.9%)	0.13	0.01–1.44				
Kicukiro	101	7 (6.9%)	0.25	0.06–1.11				
Musanze	57	13 (22.8%)	0.98	0.24–4.12				
Ngororero	17	2 (11.8%)	0.44	0.06–3.16				
Nyarugenge	195	30 (15.4%)	0.61	0.16–2.33				
**Antiretroviral treatment** 1								
Not HIV infected	323	23 (7.1%)	1		0.0007			
HIV infected, on ART	72	15 (20.8%)	3.43	1.69–6.98				
HIV infected, not on ART	168	26 (15.5%)	2.39	1.32–4.33				
HIV status unknown	11	5 (45.5%						
**Co-trimoxazole prophylaxis** 1								
Not HIV infected	323	23 (7.1%)	1		0.0004			
HIV+, on co-trimoxazole	107	14 (13.1%)	1.96	0.97–3.97				
HIV+ not on co-trimoxazole	133	27 (20.3%)	3.32	1.83–6.05				
HIV status unknown	11	5 (45.5%)						

* Farming, housewives, unemployed, laborer, vendor.

ART antiretroviral therapy, OR Odds Ratio, aOR ajusted Odds Ratio, CI Confidence Interval. PTB Pulmonary Tuberculosis.

1 On ART/co-trimoxazole: on this treatment before start of TB treatment; not on ART/co-trimoxazole: started with this treatment after starting TB treatment or not at all.

Stratified analysis by HIV status showed that among HIV-negative patients, the highest risk of death was among smear-negative PTB patients and those with EPTB. Among those co-infected with HIV, those with 3+ smear-positive PTB and those with EPTB had the highest risk of death. The interaction between HIV status and type of TB was statistically significant (p = 0.028, likelihood ratio test).

Most deaths occurred soon after the start of TB treatment: 39 (57%) had died 1 month after start of TB treatment, 54 (78%) had died by 2 months, and 63 (91%) by 3 months. Among HIV infected patients these percentages were very similar: 49%; 71%, and 90%. We did not observe important differences in timing of death between those who were treated with ART and those who were not.

### Associations between Baseline Characteristics, Adherence and Mortality

Next, we examined the effect of treatment adherence on mortality. Of 535 patients data on treatment outcome and adherence was available; 68 of them had died (13%). Those who were non-adherent were significantly more likely to die than those who were adherent (23% vs. 11%; p = 0.013). The logistic regression model was based on 517 patients with complete data. The ORs for age, type of TB and HIV status were not substantially different from the ORs in the model without adherence (although HIV status was no longer significant, OR = 1.8, 95%CI 0.9–3.3, p = 0.08); the independent effect of poor adherence was strong and very significant (OR = 3.4, 95%CI 1.4–7.8; p = 0.007).

## Discussion

In this study among TB patients in Rwanda 10% of the patients were found to be poorly adherent, defined as having missed treatment doses on >10% of the days during the treatment period. The only independent predictor of poor adherence was untreated HIV co-infection. Twenty percent of PTB+ patients failed to convert to negative smear status after 2 months of treatment. Baseline sputum smear grade and HIV infection, whether treated or not, were independent predictors for non-conversion of sputum smear at 2 months. Besides older age, type of TB (notably PTB 3+, PTB− & EPTB), and HIV infection, non-adherence was an important determinant of mortality.

According to Rwanda guidelines [13] ART was indicated in HIV patients with TB, but the timing of start of ART depended on the form of TB and the CD4 count (if EPTB or CD4 count <50 cells/mm^3^ ART should be started 15 days after start TB treatment; if CD4 count 50–200 cells/mm^3^ start after 2 months, and if CD4 count >200 cells/mm^3^ the CD4 count should be repeated after 2 months), and also on the assessment of the health care worker that the patient was likely to be adherent to ART. In this study, HIV infected patients that did not start ART before TB diagnosis were significantly more often non-adherent. The interpretation of this finding is not straightforward. Possibly these patients were not started on ART because they were reluctant to start ART, or possibly ART was delayed until after completion of TB treatment. Unfortunately we do not have CD4 data or data on treatment decisions to help interpretation.

A higher smear grade at baseline has also been identified in other studies as a predictor for non-conversion after 2 months of treatment [4], [16]–[20]. These patients have a higher bacterial load, so it may be expected that they need longer treatment until conversion occurs. The finding that HIV infection is an independent predictor for non-conversion at 2 months contrasts with results of earlier studies [16], [17], [21]. It is unlikely our results are due to high TB drug resistance levels which are not very high in Rwanda (3.9% and 9.4% for new and retreated cases respectively) [22], and HIV was found not to be associated with multidrug resistance in a systematic review of African studies [23].

HIV infection was independently associated with mortality (in the model without adherence), an expected finding. A perhaps surprising finding was that HIV patients who were treated with ART did not have a lower mortality than those who were not. This finding is hard to interpret without further clinical data or CD4 counts. Probably, those on ART were patients with more advanced HIV disease. Also, ART is not expected to have a strong impact on mortality until several months after start of ART; time on ART may have been too short for most to note an effect. With the increasing accessibility of ART in Rwanda [24], [25] the prognosis of HIV infected TB patients is now being much improved. Integration of HIV and TB services, as currently being implemented in Rwanda, will facilitate more timely ART in HIV patients, including TB patients.

Not surprisingly, older TB patients had a higher risk of death [3], [26], [27]. Older people have a higher mortality rate anyway; underlying disease as a cofactor for TB is more common in older people and may explain the increased risk of death. EPTB patients tend to have higher mortality rates than PTB+ patients [28]. The observation that smear-negative PTB patients had a high risk of death may suggest that some of these patients were misdiagnosed [29], [30] and in fact suffered from other serious cardiac or pulmonary conditions rather than TB. As expected, poor adherence was strongly and independently associated with mortality.

### Limitations

This study has some limitations. First, at the time of our study sputum smear staining in Rwanda was done by the Kinyoun cold-staining technique, which is less sensitive than the conventional Ziehl-Neelsen technique [31], [32] (introduced in Rwanda in 2009), so our data may not be comparable to those of other studies. The lower sensitivity will also have reduced the sensitivity for sputum conversion at C2 and cure (reducing the power of the study), and may have led to misclassification of smear-positive patients as smear-negative at baseline. Second, the treatment card could not be retrieved for 144 of 725 patients (20%), who tended to have more often EPTB, and had more often died. EPTB cases are normally diagnosed at district hospitals without bacteriological or histological confirmation [14] due to the complex clinical nature of their presentation. Most of these patients were subsequently transferred-out to CDTs close to their homes to start or continue treatment. Possibly the treatment cards for these patients were misplaced in the specialized departments where these patients sought additional specialized treatment for other complications including HIV [8].

The strength of our study is that it was based on routinely collected data from 110 clinics, reflecting actual health practice rather than a typical study setting. This approach comes with some inherent limitations. Important data were missing for some patients, like HIV status, sputum-smear after 2 months of intensive treatment, type of TB, or smear grade at baseline. Further, culture, drug sensitivity testing, CD4 count and HIV plasma viral load were not available. Some of the patients who were still smear positive at two months might in fact have been culture-negative, their positivity being based on non-viable mycobacteria. In a study by Su et al, this was the case in 44% of the 2 months smear positive patients [33]. Next, patients transferred-out were excluded from the adherence analysis; this may have lead to an overestimation of the proportion of adherent patients. Finally, the sample size of PTB+ cases with smear control results at 2 months was only 220, limiting the power of the analysis of smear conversion.

## Conclusions

In Rwanda HIV infection is an important independent predictor of failure of sputum smear conversion among PTB+ patients. Higher age, poor adherence and type of TB (smear 3+, smear-negative, or EPTB) are significantly associated with mortality among TB patients, and HIV infection has a near-significant effect. Improving adherence is gaining even more importance, now that increasing proportions of TB patients will be taking both TB treatment and ART. Operational research is needed to explore innovative approaches that may improve TB treatment adherence.
